# Strategies to Optimise Oncolytic Viral Therapies: The Role of Natural Killer Cells

**DOI:** 10.3390/v13081450

**Published:** 2021-07-26

**Authors:** Elaine Y. L. Leung, Iain A. McNeish

**Affiliations:** 1 Institute of Cancer and Genomic Sciences, College of Medical and Dental Sciences, University of Birmingham, Birmingham B15 2TT, UK; 2 Ovarian Cancer Action Research Centre, Department of Surgery and Cancer, IRDB Building, Imperial College London, London W12 0NN, UK

**Keywords:** oncolytic virus, NK cells, immunotherapy, gene therapy, neoplasms

## Abstract

Oncolytic viruses (OVs) are an emerging class of anti-cancer agents that replicate selectively within malignant cells and generate potent immune responses. Their potential efficacy has been shown in clinical trials, with talimogene laherparepvec (T-VEC or IMLYGIC^®)^ now approved both in the United States and Europe. In healthy individuals, NK cells provide effective surveillance against cancer and viral infections. In oncolytic viral therapy, NK cells may render OV ineffective by rapid elimination of the propagating virus but could also improve therapeutic efficacy by preferential killing of OV-infected malignant cells. Existing evidence suggests that the overall effect of NK cells against OV is context dependent. In the past decade, the understanding of cancer and OV biology has improved significantly, which helped refine this class of treatments in early-phase clinical trials. In this review, we summarised different strategies that have been evaluated to modulate NK activities for improving OV therapeutic benefits. Further development of OVs will require a systematic approach to overcome the challenges of the production and delivery of complex gene and cell-based therapies in clinical settings.

## 1. Introduction

### 1.1. Oncolytic Viruses (OV) as an Emerging Class of Immunotherapy

Oncolytic viruses (OVs) are an emerging class of anti-cancer agents, with proven efficacy in randomised clinical trials and potential to produce durable treatment response [1,2,3]. The ideal OV preferentially infects and replicates in cancer cells, inducing cell death to release newly synthesised virions that consequently infect neighbouring cells and promote further cancer cell death (direct oncolysis). However, it is increasingly recognised that the therapeutic efficacy of OVs is dependent less on tumour cell lysis than on establishing anti-tumour immunity [4,5,6].

OVs are usually modified to promote tumour selectivity, which can be driven by a number of factors. Firstly, receptor-mediated and virus-specific mechanisms determine viral entry. Some viruses may preferentially eliminate malignant cells intrinsically [7]. For example, measles viral entry receptors (e.g., CD46) are highly expressed by many tumour cells [8]. OVs can also be engineered to retarget cell entry specifically into cells bearing cell surface receptors upregulated on malignant cells, such as EGFR [9,10,11]. Secondly, tumour molecular characteristics (e.g., the presence of *RB1* mutations) and the high metabolic and replicative activities exhibited by tumour cells support the preferential replication of OVs within tumour cells [12,13]. Thirdly, the immune responses induced by OV infection could both hinder viral spread and concurrently promote anti-tumour immunity [4,5,6]. OVs are often engineered to augment anti-tumoural immune responses. For example, talimogene laherparepvec (T-VEC or IMLYGIC^®^) [2,14] is an oncolytic herpes simplex virus (HSV) engineered to express human granulocyte macrophage colony-stimulating factor (hGM-CSF) to augment anti-tumoural T-cell responses [15,16]. T-VEC has demonstrated a significantly higher durable response rate (19.0% vs. 1.4%; *p* < 0.0001) and improved overall survival (23.3 months and 18.9 months; *p* = 0.049) compared to GM-CSF alone in patients with melanoma who were not amenable to surgical resection [2,17].

### 1.2. The Potential of Natural Killer (NK) Cells to Enhance OV Efficacy

T-VEC remains the only OV approved by the Food and Drug Administration (FDA) of the United States, the National Institute for Health and Care Excellence (NICE) of the United Kingdom and the European Medicines Agency (EMA) for the treatment of advanced melanoma. The regulatory approval of T-VEC and the fact that T-VEC expresses hGM-CSF, an important haematopoietic growth factor and immune modulator, have incentivised research and further clinical trials on improving the efficacy of OV by enhancing anti-tumoural immune responses.

Although Natural Killer (NK) cells mediate rapid viral clearance that could render OV less effective, their preferential lysis of virally infected cells could also promote the therapeutic response. Conflicting evidence has been reported on the role of NK cells in the overall efficacy of OVs with both enhancement [18,19,20,21] and reduction of anti-tumour activity [22,23,24].

In this review, we provide a brief overview on the role of NK cells in the context of OV and the current strategies to improve OV efficacy via NK cell modulations. In addition, we provide concise summaries and key references on NK cell development and the determinants of NK responses for those less familiar with this immune cell population

## 2. Natural Killer (NK) Cell Responses in the Context of OV

### 2.1. NK Cell—A Member of the Innate Lymphoid Cell Family

**Figure 1:**
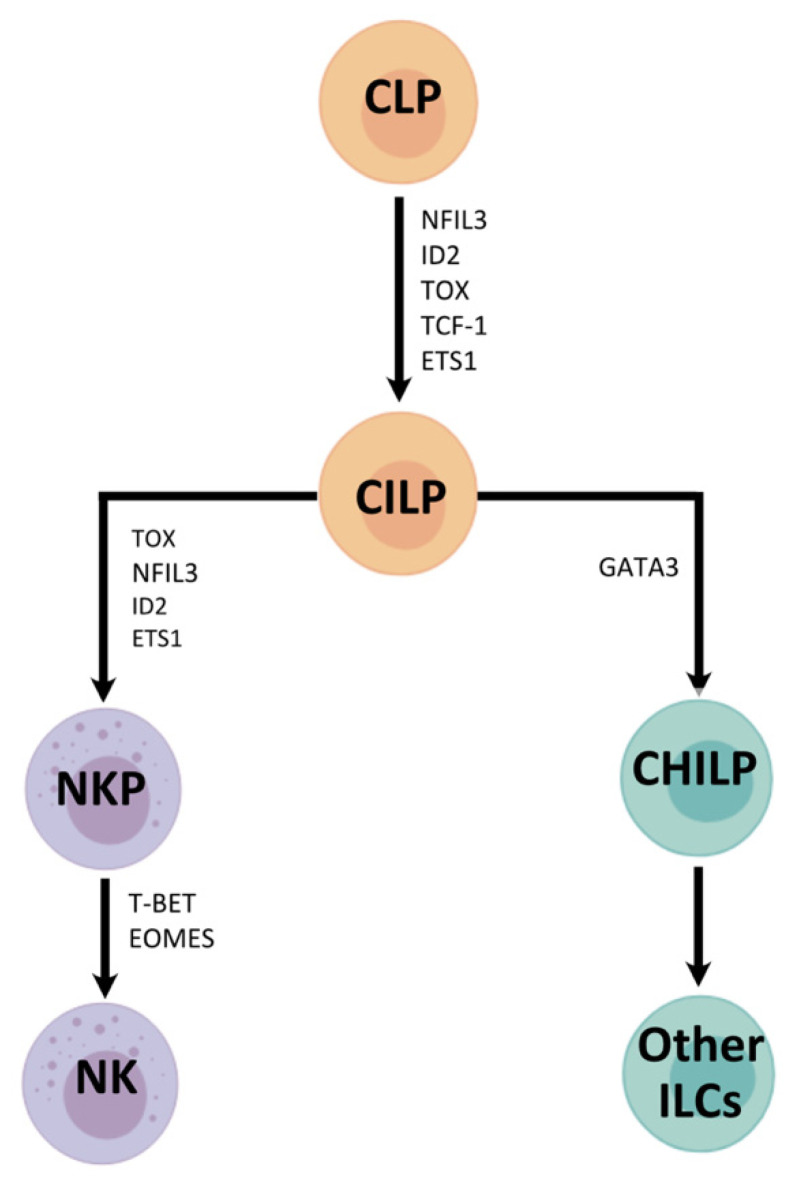
NK cell development with summaries of the transcription factors required at each stage of differentiation [28]. NK cells originate from common lymphoid progenitors (CLPs), which differentiate into CILPs (common innate lymphoid progenitors). CILPs then differentiate into NK cell precursors (NKP) or common helper innate lymphoid progenitors (CHILPs). The former give rise to NK cells, and the latter develops into other ILCs. NFIL3 = nuclear factor IL-3 induced; ID2 = inhibitor of DNA binding 2; TOX = thymocyte selection-associated high mobility group box protein; TCF-1 = T cell factor 1; ETS1 = avian erythroblastosis virus E26 homolog-1; GATA3 = GATA binding protein 3; T-bet = T-box transcription factor; Eomes = Eomesodermin.

NK cells are circulating cytotoxic cells that have the ability to kill virus-infected normal cells and tumour cells. They also demonstrate a high level of perforin expression upon activation [25]. NK cells are a member of the innate lymphoid cell (ILC) family, a heterogenous group of cells derived from common lymphoid progenitors that lack adaptive antigen receptors [26,27,28,29]. The nomenclature of ILCs can be confusing as it has evolved since the proposed uniform nomenclature in 2013 by Spits and co-workers [28]. Currently, five major groups of ILCs have been defined based on transcriptional factors required during their development and their cytokine production patterns (Figure 1). They include NK cells, Group 1-3 ILCs (ILC1-3) and lymphoid tissue-inducer (LTi) cells. Initially classified as a member of ILC1 [26], which have overlapping phenotypical markers and also secrete IFNγ in a T-bet-dependent manner, NK cells are now recognised as a discrete group of ILC, and are distinguished from ILC1s by the requirement for the T-box factor EOMES for their development from NK cell precursors [29].

### 2.2. The Importance of NK Cells in Anti-Viral and Anti-Tumoural Defence

The importance of NK cells as our natural anti-viral defence is apparent in the study of patients with primary NK deficiencies. Five classical NK deficiencies (also known as developmental NK deficiencies) have been described [30]. They are defined by CD56^+^ CD3^−^ NK cell counts ≤ 1% of total peripheral lymphocytes. Classical NK deficiencies are linked to different impact on NK subset quantity and functions, as well as clinical effects [30]. The hallmark presentation of classical NK deficiency is HSV infections (60% of reported cases; often recurrent and severe) [31]. However, with the exception of malignancies associated with oncogenic viruses (e.g., EBV-associated cancers), classical NK deficiencies have not been consistently associated with higher risk of other cancers in the small cohorts of patients reported [31]. With the advances of medical treatment and patients with primary immunodeficiencies surviving longer, the impact of these rare diseases on the risk of cancers in older patients may become more apparent in the future [30].

It has long been recognised that NK cells are also important in cancer. The early in vivo NK depletion experiments (with anti-asialo-GM1 or anti-NK1.1 antibodies) in methylcholanthrene (MCA)-induced fibrosarcoma and transplantable murine models two decades ago provided initial evidence of the importance of NK in controlling tumour growth [32,33]. These results were subsequently confirmed in genetically modified murine models lacking natural killer cell p46-related proteins (NKp46 or NCR1) [34,35]. In humans, studies have shown that the cytotoxicity of peripheral blood NK cells in patients with cancer are lower than in healthy controls [36,37,38,39,40,41,42]. Low levels of NK cells have also been associated with higher cancer incidence [43], risks of recurrence [37,44,45] and poorer survival [37,44,45] in multiple cancer types.

The clear importance of NK cells in controlling both anti-viral and anti-cancer responses indicated that modulation of NK cell activities has the potential to promote the OV efficacy. OVs have been developed from a range of different virus families [4], including herpesvirus [46], reovirus [47], polio virus [48], parvovirus [49], vaccinia [50] and adenovirus [51]. These viruses have vastly different structures, lifecycles, tropisms and toxicities, and induce variable immune responses that could influence the efficacy in different settings [52,53]. Understanding the determinants of NK cell responses in the context of OVs is therefore crucial to improve this class of treatments.

### 2.3. Determinants of NK Cell Response in the Context of OV

NK activities are modulated by different cell surface receptors (Table 1), which can activate or inhibit NK functions [29]. Importantly, NK cells have HLA class I specific receptors, primarily killer Ig-like receptors (KIRs), which can activate or inhibit NK cells [54,55]. Less studied non-KIR NK cell receptors, including Natural Killer Group 2 (NKG2) receptors, natural cytotoxicity receptors (NCRs) and immunoglobulin-like transcripts (ILTs), are increasingly recognised to have potential roles in cancer immunotherapies. These are reviewed elsewhere [56,57]. Healthy cells express HLA class I molecules to generate inhibitory signals to avoid autoreactivity via inhibitory KIRs or other inhibitory NK receptors (e.g., NKG2A). Virus-infected cells and tumours often demonstrate downregulation of HLA class I molecules, which will act to reduce inhibitory NK signals and trigger NK activation and targeted cell killing [54,55].

NK cells are one of the most important groups of lymphocytes that can induce antibody-dependent cellular cytotoxicity (ADCC) against opsonized cells via the Fc receptor CD16 (FcγRIIIa). ADCC has been exploited to develop treatments for cancers and other diseases. For example, the anti-CD20 monoclonal antibody rituximab is widely used for the treatment of non-Hodgkin lymphoma and autoimmune diseases [58]. Similarly, anti-viral humoral responses elicited by OVs can lead to ADCC against viral-infected tumour cells, which have been explored to augment OV in preclinical settings [59,60].

NK cells also express cytokine receptors, which orchestrate both innate and adaptive immune responses through cytokine and chemokine production. For example, a recent report suggested NK cells recruit dendritic cells (DC) into murine solid tumours via CCL5, XCL1 and XCL2, and both chemokine expression and the presence of DC were associated with improved survival in selected cancer types [61]. DC can engage with NK cells via natural killer cell p30-related protein (NKp30/NCR3), which induces NK expression of IFNγ and further promotes DC maturation [62,63].

OVs have been shown to induce NK recruitment, activation and ultimately elimination of the virus-infected host cells [54,64]. This recruitment could lead to rapid clearance of OVs and attenuate their efficacy but could also act as part of OV anti-tumoural responses [65]. The majority of studies using reovirus [19,21,45], maraba virus [66] and adenovirus [67] as vectors demonstrated that oncolytic virus efficacy was enhanced by eliciting an NK cell-mediated anti-tumour response in both murine and human preclinical models. For example, the recognition of oncolytic reovirus by DCs led to secretion of chemokines CCL2, 3, 4, 5, 7, 8, 11 and CXCL10 [68], which induced NK chemotaxis and augmented NK cytotoxicity against infected tumour cells. Our recent work on oncolytic adenoviral therapies showed that infected tumour cells induced contact-dependent NK activation and augmented anti-cancer cytotoxicity [67]. However, other reports have suggested that NK cells could also reduce the efficacy of oncolytic HSV in murine glioblastoma [23,24,69] and vesicular stomatitis virus (VSV) in rat hepatocellular carcinoma models [70]. In addition, HSV reduces NK activities via NKp30 and NKp46 [71], which could be reversed by TGF-β treatment [24].

The balance between regulatory and anti-viral roles of NK cells is likely to be context dependent. Variations in treatment response and survival after OVs have also been associated with intrinsic patient factors, such as Fc-gamma receptor genetic polymorphism [72]. Evaluating the mechanisms underpinning why some patients achieve a significantly better response after specific OVs is now possible with the routine clinical use of selected OVs [73]. Different strategies to optimise the synergy between the anti-tumoural immune responses and the direct cytotoxic effects of OVs in different clinical circumstances have also been explored.

Here, we summarise strategies that have been evaluated to exploit NK cell responses to augment the anti-tumoural effects of OVs.

## 3. Strategies That Exploit NK Cell Response to Improve OV Efficacies

### 3.1. Pharmacological Modulation of NK Response

Combination therapies are being actively explored to modulate NK responses to improve OV efficacies, either by specifically targeting NK receptor–ligand interactions or by influencing the broader tumour microenvironment. For example, expression of T-cell immunoglobulin and ITIM domain protein (TIGIT), but not the checkpoint molecules CTLA-4 and PD-1, was associated with NK cell exhaustion in cancer [74]. We recently showed an upregulation of TIGIT on primary human NK cells after co-culture with ovarian cancer lines [67]. In addition, TIGIT blockade was previously shown to prevent NK cell exhaustion and promote NK-dependent anti-tumoural responses in murine models [74], whilst we showed that TIGIT blockade could augment the NK cytotoxicity of oncolytic adenovirus-infected human ovarian cancer cells [67]. The combination of OVs with a variety of licensed immune checkpoint inhibitors has been investigated in multiple clinical trials, which were recently summarised elsewhere [75]. These combinations can stimulate NK and other immune cells bearing the specific immune checkpoints.

NK-associated improvements in OV efficacy have also been demonstrated by other combinations that aimed to influence the broader tumour microenvironment. Inhibition of innate immune responses, e.g., neutralisation of virus-induced chemokines, enhanced VSV efficacy [20,76]. The use of the proteasome inhibitor bortezomib [69] and TGF-β [24] augmented NK cytotoxicity against oncolytic-HSV-infected tumours in vitro and in vivo. More recently, combining the histone deacetylase inhibitor valproic acid with T-VEC was shown to promote viral replication, expression of NK-activating NKG2D ligands (MICA/B and ULBP2/5/6) and preclinical effectiveness in melanoma [77].

### 3.2. Manipulation of OV to Augment NK Activities

OVs have been directly engineered to express relevant NK receptors to augment OV activities. As NK cells have been shown to impede oncolytic HSV activities [23,24,69], oncolytic HSV was engineered to express E-cadherin, an adhesion molecule and a ligand for NK inhibitory receptor KLRG1, resulting in improved survival in mouse models of glioblastoma [78]. Arming HSV with secreted chimeric molecules to induce innate immune cell killing of infected tumours has also been explored [79]. In contrast, as NK cells were shown to augment oncolytic vaccinia virus activities [4], engineering expression of a monoclonal antibody against TIGIT was able to enhance preclinical effectiveness [80]. Similarly, an adenovirus expressing fusion protein PD-1/CD137L demonstrated anti-tumoural response in murine hepatocellular carcinoma [81].

OVs have also been manipulated to secrete selected cytokines [82,83,84] and chemokines [85] to augment NK responses and improve OV efficacy. Engineered oncolytic HSV [82] and vaccinia virus [83] that secrete interleukin(IL)-15, a cytokine that preferentially stimulates NK cell and CD8 T cell function and proliferation, were both shown to improve OV effectiveness compared to unmodified viruses, with [83] and without [82] combination with immune checkpoint inhibitors. Additional transgenes have also been inserted to create an oncolytic HSV (VG161) that enabled the concurrent expression of IL-12, IL-15 and IL-15 receptor alpha subunit, and showed improved effectiveness in two syngeneic in vivo models [84]. CCL5 is a key chemokine that induces chemotaxis of NK and other immune cells to inflammatory sites and cancer, and CCL5-expressing oncolytic vaccinia virus was shown to promote NK cell infiltration and anti-tumour activity, a xenograft model of colonic cancer [85]. These approaches aim to minimise the side effects of systemic administration of cytokines and chemokines by targeted expression within OV-infected cells, although data on their clinical efficacy and toxicity profile remain limited.

### 3.3. Adoptive Transfer of NK Cells

The potential benefits of NK cell-based therapies over T-cell-based therapies include fewer graft-versus-host reactions and the possibility to develop off-the-shelf products [86,87]. A variety of NK cell products (e.g., autologous NK cells, haploidentical NK cells, chimeric antigen receptor NK cells, stem cell-derived NK cells and NK cell lines) have been explored as NK cell-based therapies [86,87]. There is also an increasing number of methods for enhancing NK activity in vivo as reviewed elsewhere [86,87]. There are currently few direct comparative studies of different choices of NK cell sources and different in vivo NK enhancement strategies [86].

Adoptive transfer of NK in patients receiving OVs has been explored to enhance treatment response. The combination of CCL5-expressing oncolytic vaccinia virus and CCR5-engineered NK cells has been explored to further enhance therapeutic effectiveness [85]. The addition of adoptive NKG2D-positive cells has improved the effectiveness of oncolytic measles viral therapy in a murine hepatocellular carcinoma model [88]. With the emergence of chimeric antigen receptor (CAR) immune cell therapies, CAR-NK cells have been administrated intracranially in a murine model of breast cancer brain metastases in combination with oncolytic HSV [89]. Ex vivo activated and expanded NK cells were also evaluated in paediatric sarcomas in vitro [90].

### 3.4. Other Strategies

To overcome the detrimental effects of virus-neutralizing antibodies on OV efficacy, a novel strategy has been proposed to use a bifunctional molecule with a tumour-specific ligand and the adenovirus hexon domain DE1 against anti-adenoviral antibodies to re-direct adenoviruses to the tumour and induce ADCC [59]. The combination of this bifunctional molecule with oncolytic adenovirus has showed improved outcomes in subcutaneous murine models compared to oncolytic adenovirus alone. Further in vivo immune cell depletion suggested the therapeutic benefits were associated with NK cell-dependent CD8 T-cell activities [59].

Another emerging strategy involves the use of bispecific immune cell engagers [75]. These bispecific molecules are able to link two specific single-chain antibody variable fragments (scFvs) via a ligation peptide. Typically, one of the scFvs specifically binds to a tumour antigen on cancer cells, while the other binds to an immune activator on the target immune cells. These novel molecules could be incorporated with OV therapies as combination therapy (e.g., with Blinatumomab, a bispecific T cell engager [BiTE]) or by developing OVs that encode these bispecific molecules [91]. The use of BiTEs with OV has only recently been explored in preclinical studies [91,92]. With the development of multifunctional NK cell engagers (NKCEs) [93], it is plausible that these could be also used to augment OV efficacy.

### 3.5. Considerations for Future Research

**Figure 2:**
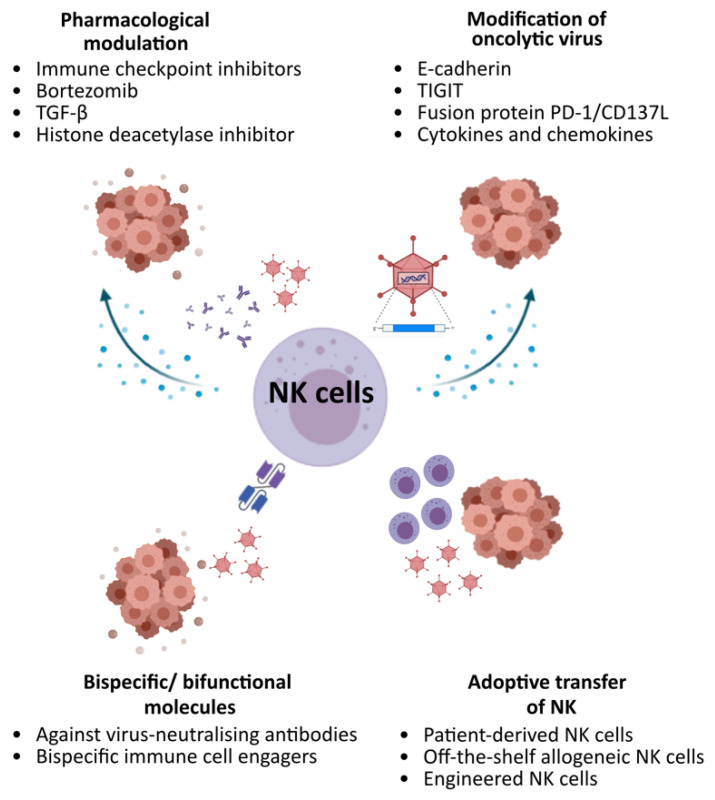
Potential strategies to augment OV activities via NK cells.

The majority of strategies explored so far modulate NK cell responses by pharmacological combinations, further engineering of OV and/or adding NK cells by adoptive transfer (Figure 2). However, the inter-relationships between OV, NK and other facets of the host immune response and tumour microenvironment are complex. Strategies explored often induce other immunological consequences on other immune cell populations, and not all augment OV efficacy. Fine tuning these strategies by considering different interrelated factors, including the temporal effects (i.e., when to administer), types of OVs, sites and types of malignancies, would be crucial to maximise therapeutic benefits.

**Figure 3:**
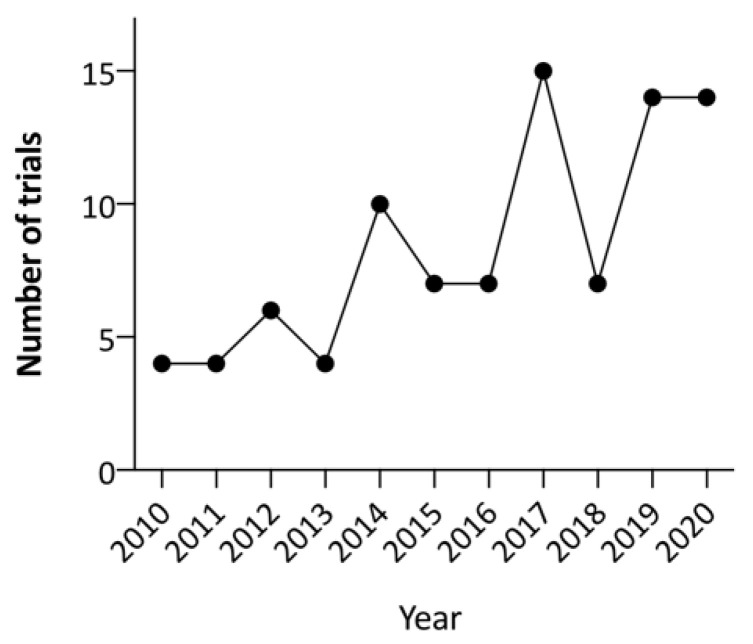
The number of oncolytic virus clinical trials commenced each year since 2010 and registered at ClinicalTrials.gov.

Although most strategies explored have only been evaluated in preclinical settings, there has been a significant increase in the number of new OV trials over the past decade. A search of the term ‘neoplasms’ combined with ‘oncolytic virus’ in the ClinicalTrials.gov database in late April 2021 found 118 OV trials, the majority (100/118; 84.7%) of which commenced after 2010 (Figure 3). However, delivery of complex gene and cell-based therapies poses challenges compared to traditional pharmacological treatments [94]. Significant translational barriers remain to produce and deliver these therapies for patients, both within and outside clinical trial settings. In addition to identifying when and which OV should be given for each type of cancer, the mode of delivery and the optimal use of combination therapies both remain to be determined. As the costs of omics technologies have reduced over time, with the development of different clinically approved genomic panel tests, routine molecular profiling of cancers by proteomics or genomics methods have the potential to help develop predictive biomarkers to further improve trial design and clinical applications of OV in the future [95].

## 4. Conclusions

Although a small number of OVs have now been approved for clinical use in selected cancers such as advanced melanoma, their use in other oncological settings remains unclear. NK cells are the prime immune cell population to control viral infections in humans, but their ability to eliminate viral-infected cells rapidly could lead to opposing impacts on the therapeutic outcomes of oncolytic viral therapies. Existing evidence suggests the overall effect of NK cell response on the efficacy of OV is context dependent. Different strategies have been evaluated for improving OV by NK cell modulation. Further development of OVs for clinical use requires not only understanding of the biology of cancers and OV but also overcoming the challenges of the production and delivery of complex novel therapies in clinical settings.

## Figures and Tables

**Table 1:** NK activating, inhibitory and cytokine receptors in mice and humans [29].

Activating	Inhibitory	Cytokine
Activating KIRs NKp46 NKp44 (human only) NKp30 (human only) NKG2C NKG2D NKG2E CD16 2B4 DNAM1 Ly49D, H, L (mouse only)	Inhibitory KIRs TIGIT CD96 LAG3 TIM3 PD1 KLRG1 CD161 NKG2A NKG2B Ly49A, B, C, E, G, Q (mouse only)	IL-2R IL-4R IL-10R IL-12R IL-15R IL-18R IL-21R TGF-β

## Data Availability

Not applicable.
